# Carvacrol as a Stimulant of the Expression of Key Genes of the Ginsenoside Biosynthesis Pathway and Its Effect on the Production of Ginseng Saponins in *Panax quinquefolium* Hairy Root Cultures

**DOI:** 10.3390/ijms25020909

**Published:** 2024-01-11

**Authors:** Ewa Kochan, Monika Sienkiewicz, Dagmara Szmajda-Krygier, Ewa Balcerczak, Grażyna Szymańska

**Affiliations:** 1 Department of Pharmaceutical Biotechnology, Medical University of Lodz, Muszynskiego 1, 90-151 Lodz, Poland; 2 Department of Pharmaceutical Microbiology and Microbiological Diagnostics, Medical University of Lodz, Muszynskiego 1, 90-151 Lodz, Poland; monika.sienkiewicz@umed.lodz.pl; 3 Department of Pharmaceutical Biochemistry and Molecular Diagnostics, Medical University of Lodz, Muszynskiego 1, 90-151 Lodz, Poland; dagmara.szmajda@umed.lodz.pl (D.S.-K.); ewa.balcerczak@umed.lodz.pl (E.B.)

**Keywords:** carvacrol, *FPS*, *SS*, *SD* genes, ginsenosides, hairy roots, real-time PCR

## Abstract

The accumulation of ginsenosides (triterpenic saponins) was determined in *Panax quinquefolium* hairy root cultures subjected to an elicitation process using carvacrol at 5, 10, 25, 50, 100, 250, and 500 μM concentrations during 24 and 72 h exposure. This study was the first one in which carvacrol was applied as an elicitor. The content of eight ginsenosides, Rb1, Rb2, Rb3, Rc, Rd, Rg1, Rg2, and Re, was determined using HPLC analysis. Moreover, the quantitative RT-PCR method was applied to assess the relative expression level of farnesyl diphosphate synthase, squalene synthase, and dammarenediol synthase genes in the studied cultures. The addition of carvacrol (100 μM) was an effective approach to increase the production of ginsenosides. The highest content and productivity of all detected saponins were, respectively, 20.01 mg∙g^−1^ d.w. and 5.74 mg∙L^−1^∙day^−1^ after 72 h elicitation. The production profile of individual metabolites in *P. quinquefolium* cultures changed under the influence of carvacrol. The biosynthesis of most examined protopanaxadiol derivatives was reduced under carvacrol treatment. In contrast, the levels of ginsenosides belonging to the Rg group increased. The strongest effect of carvacrol was noticed for Re metabolites, achieving a 7.72-fold increase in comparison to the control. Saponin Rg2, not detected in untreated samples, was accumulated after carvacrol stimulation, reaching its maximum concentration after 72 h exposure to 10 μM elicitor.

## 1. Introduction

Plant secondary metabolites (PSM) regulate numerous physiological processes such as plant development and plant adaptation to the environment. PSM often play an important role in plant defense against pathogens and abiotic stress factors. Some of these metabolites may constitute a chemical barrier, while others demonstrate a function in signaling processes related to plant protection [1,2]. In addition, secondary metabolites, i.e., alkaloids, glycosides, phenolics (e.g., simple phenolics, coumarins, tannins, lignans, and flavonoids) saponins, terpenes, and essential oils, have therapeutic potential, showing multidirectional pharmacological activities [3,4]. Ginsenosides—triterpene saponins isolated from ginseng—are an example of PSM, whose biological activity has been confirmed by many studies. Experiments, conducted both in vitro and in vivo, proved the effectiveness of ginseng extracts on the body’s metabolism and psychophysical performance restoring body homeostasis. Ginseng affects the functions of organs and systems (nervous, hormonal, cardiovascular, immune, and digestive) at the cellular level [5]. Saponins, contained in ginseng, regulate the permeability of cell membranes. They modify cell membranes’ structure by reacting with specific proteins, and, thus, they can influence the activity of membrane receptors, enzymes, and ion channels [6].

**Figure 1:**
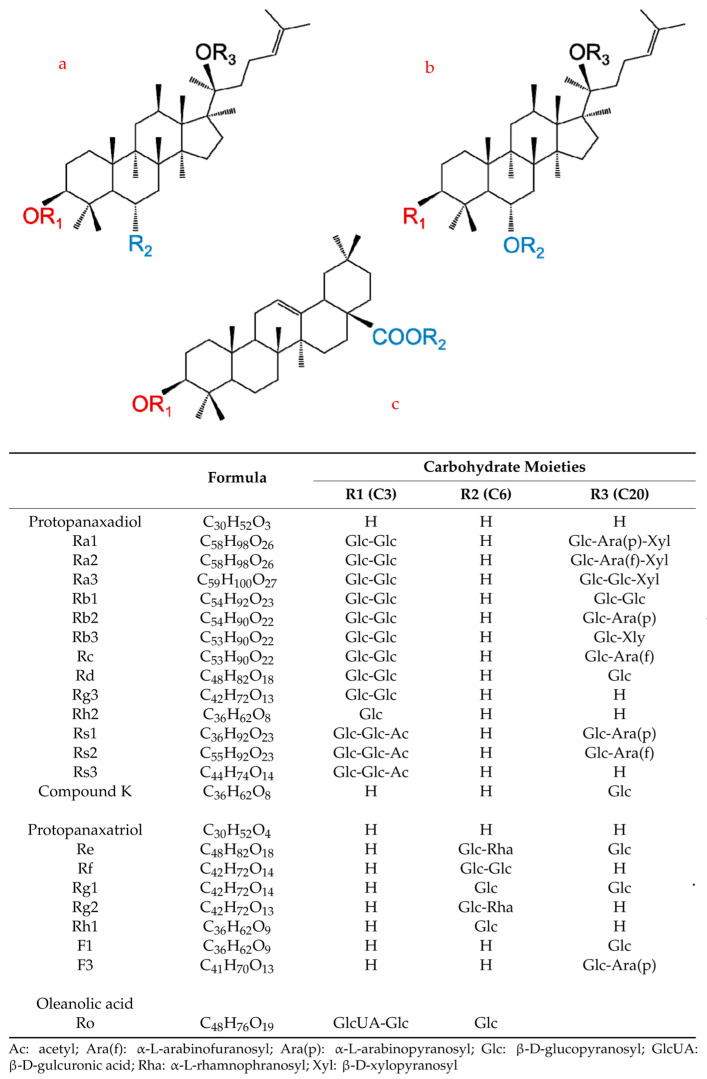
Chemical structures of three ginseng saponin types: (**a**) protopanaxadiol type (PPD); (**b**) protopanaxatriol type (PPT); and (**c**) oleanolic acid type [7].

Ginsenosides, being glycoside compounds, consist of a non-sugar part—aglycone—and a sugar chain or chains. There are three types of aglycones in the chemical structure of ginsenosides: aglycones of the tertacyclic dammarane type (the most important ones include derivatives of 20(S)-protopanaxadiol (PPD) and 20(S)-protopanaxatriol (PPT)), aglycones of the pentacyclic oleanolic acid type, and aglycones of the tetracyclic ocotilol type. The sugar part of saponins most often includes hexoses (glucose and galactose), 6-deoxyhexoses (furanose and rhamnose), pentoses (arabinose and xylose), and uronic acids (glucuronic acid) [7]. They usually occur in a cyclic form and form hemiacetal bonds with aglycone. Ginsenosides, consisting of 17 carbon atoms in a four-ring structure with various sugar residues attached to the C-3, C-6, and C-20 positions, are mostly glycosidic derivatives of dammaran (Figure 1). The main ginsenosides are divided into two types: 20(S)-protopanaxadiol derivatives and 20(S)-protopanaxatriol derivatives. PPD derivatives include metabolites such as Rb1, Rb2, Rb3, Rc, Rd, Rg3, and Rh2. These are referred to as ginsenosides of the Rb group (RbG). PPT derivatives include panaxosides Re, Rf, Rg1, Rg2, Rh1, F1, and F3. In contrast, these compounds are referred to as ginsenosides of the Rg group (RgG) [7]. In addition, several rare ginsenosides have been identified, such as ocotillol saponin, represented by F11 (24-R-pseudoginsenoside) or majonoside R2, and pentacyclic oleanate saponin Ro [8,9].

**Figure 2:**
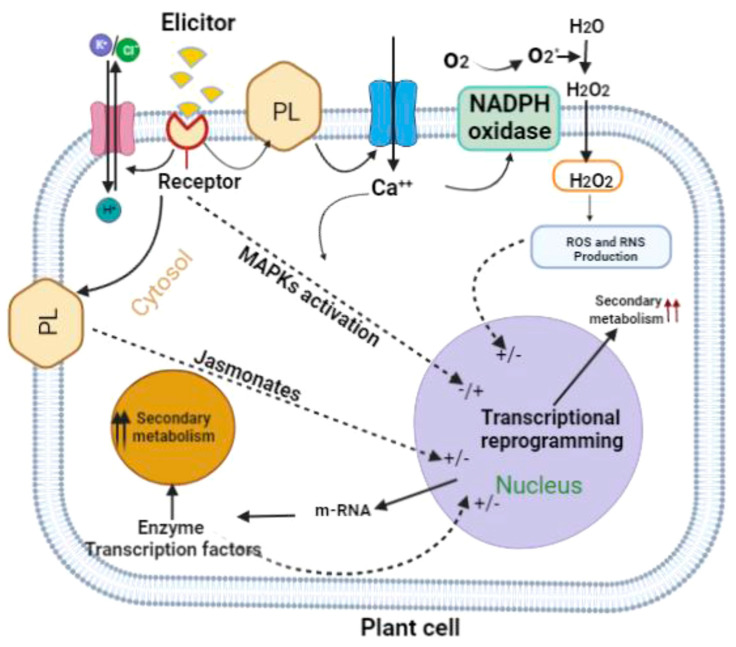
Scheme of the general mechanism action of elicitors [11]. PL—phospholipase; ROS—reactive oxygen species; RNS—reactive nitrogen species; MAPKs—mitogen-activated protein kinases.

The biosynthesis of secondary metabolites can be stimulated by applying elicitors. Elicitors are chemical compounds from various sources or physical factors that can induce defensive reactions in plants, leading to enhanced synthesis and the more effective accumulation of secondary metabolites [10]. The exact molecular mechanism of elicitors that affects cell metabolism is still the subject of research by many scientific centers (Figure 2).

It is considered that, at first, the elicitor connects to the receptor located on the surface of the cell, by means of which the cell recognizes the type of signal and generates a response. This process is followed by a number of subsequent changes: the reversible phosphorylation and dephosphorylation of proteins associated with the membrane, cytosol, and Ca^2+^ ion channel and the efflux of chloride and potassium ions, which, in turn, results in the extracellular alkalinization and acidity of the cytoplasm. These changes activate various signaling pathways, such as the mitogen-activated protein kinase (MAPK) pathway, and lead to the activation of NADPH oxidase and the accumulation of reactive oxygen and nitrogen species (ROS and RNS). Next, the expression of late defense response genes and the production of jasmonate occur. Finally, the accumulation of secondary metabolites takes place [11,12].

The application of elicitors is one of most effective methods to increase the production of secondary metabolites in hairy root cultures. These cultures are able to accumulate very important pharmaceutical compounds: tropane alkaloids (scopolamine and hyoscyamine), indole alkaloids, cardenolide glycosides, polyacetylenes, thiophenes, anthraquinones, taxol, and others. Moreover, the profiles of these metabolites are the same as in mother plants, and their level can be higher than that of the parent plant or of other types of in vitro plant tissue cultures [13,14,15,16,17]. Biotic and abiotic elicitors were also applied to enhance ginsenoside production in *Panax ginseng* hairy root cultures. According to scientific data, the external application of jasmonic acid and its methyl derivative significantly improved ginsenoside production in hairy root cultures of *P. ginseng* growing both in flasks and a bioreactor [18,19,20,21]. Similar effects were noticed after peptone treatment [20]. Other elicitors such as vanadyl sulphate, phenol derivatives (caffeic acid and catechine), fucoidan, chitosan, and gum karaya decreased the ginseng saponin content [19,20,21]. Yu et al. [22] examined the influence of different types of light on ginsenoside accumulation. Fluorescent light stimulated ginsenoside production, whereas metal halide, blue, red, and blue plus red light reduced ginseng saponin content in hairy root cultures of *P. ginseng* growing in a flask bubble bioreactor. Liang et al. [23] reported that the level of total ginsenosides was enhanced under Tween treatment, and most of these metabolites were released into the medium. Moreover, it was stated that the level of dammarenediol synthase, being one of the enzymes involved in ginsenoside biosynthesis, was up-regulated at both the gene expression and enzyme activity levels. Tween elicitation also slightly contributed to the up-regulation of the expression of genes *CYP716A47* and *CYP716A53v2*, encoding Cyt P450 enzymes and catalyzing the formation of protopanaxadiol from dammarenediol and protopanaxatriol from protopanaxadiol, respectively. Other authors demonstrated that the treatment of *P. ginseng* hairy root cultures with methyl jasmonate (MJ) also resulted in the increased expression of genes and coding enzymes involved in the ginsenoside pathway. In consequence, the increased activity of farnesyl diphosphate synthase (FPS), squalene synthase (SS), and squalene epoxidase1 (SQE1) and enhanced ginsenoside accumulation were observed [24,25,26,27]. Transformed root cultures were also obtained from another species of ginseng—*P. quinquefolium*—and several elicitors have, so far, been used to promote ginsenoside biosynthesis in these cultures [28]. The properties of well-known elicitors such as yeast extract, abscisic acid, and methyl jasmonate were studied [29,30,31]. Furthermore, our team attempted to find new stressors that would increase the accumulation of ginsenosides in the studied cultures. We turned our interest to essential oils. Their high, multi-directional biological activity [32] gave rise to the idea that they may also affect plant metabolism. We showed that oil components such as linalool and *trans*-anethole can enhance the biosynthesis of ginsenosides and lead to the accumulation of ginseng saponin at a level comparable to that observed in roots grown in field conditions [33,34]. These results inclined us to search for other essential oil ingredients to obtain even higher-efficiency saponin synthesis in the studied cultures.

This research is the first to analyze the influence of carvacrol on ginsenoside production in hairy root cultures of *P. qunquefolium*. The optimum carvacrol concentration and time of elicitation were estimated for maximum saponin production in the studied cultures. Moreover, the additional novelty of this paper was that the verification of carvacrol can act on the molecular level and regulate the expression of selected genes encoding enzymes engaged in ginseng saponin biosynthesis. Thus, the effect of carvacrol on the expression of *FPS*, *SS*, and *dammarenediol synthase* (*DS)* was assessed, with the use of quantitative RT-PCR experiments executed at different time exposures and different elicitor concentrations. The role of carvacrol in the accumulation of ginseng saponins or other secondary metabolites has not been documented in any in vitro plant culture.

## 2. Results

This study investigated the effect of carvacrol on the expression levels of genes coding three main enzymes, farnesyl diphosphate synthase, squalene synthase, and dammarenediol synthase, involved in ginsenoside biosynthesis in hairy root cultures of *P. quinquefolium* cultivated in shaken flasks. Additionally, in these cultures, the content of eight ginsenosides (Rb1, Rb2, Rb3, Rc, Rd, Rg1, Rg2, and Re) was examined after carvacrol treatment at different concentrations and during different elicitation times.

### 2.1. Effect of Carvacrol Treatment on Expression Level of Studied Genes in Hairy Roots of P. quinquefolium

**Figure 3:**
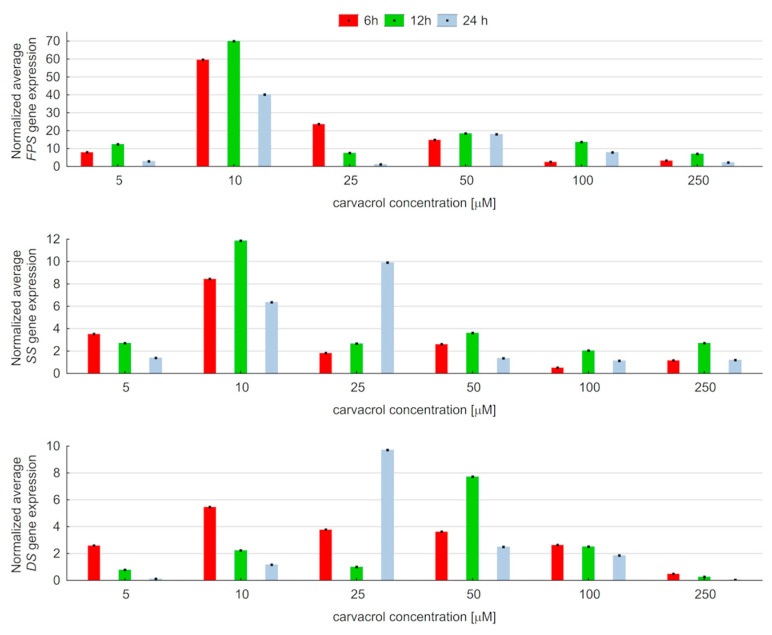
Normalized expression of *FPS*, *SS*, and *DS* genes in hairy root cultures of *P. quinquefolium* elicited with carvacrol.

The expression level of three genes, *FPS, SS*, and *DS*, involved in ginsenoside biosynthesis was estimated by RT-PCR. An analysis was carried out after 6, 12, and 24 h of treatment with carvacrol at 5, 10, 25, 50, 100, 250, and 500 µM concentrations. It was observed that the elicitation process leads to the overexpression of *FPS*, *SS*, and *DS* genes. However, the profile of up-regulation depends on the carvacrol concentration and the time of its treatment (Figure 3). Moreover, it was found that carvacrol, at all the used concentrations, brought about an increase in the transcript abundance of farnesyl diphosphate synthase, squalene synthase, and dammarenediol synthase. After 6 h of elicitation, the dynamics of expression changes were similar for all tested genes. In this condition, the maximum level of gene expression, different for each one, was noted for 10 µM carvacrol. It was also observed that a longer-term (12 h) application of carvacrol at a 10 µM concentration showed the strongest overexpression of two studied genes: *FPS* and *SS.* Then, the transcripts level of *FPS* was 70-fold higher in comparison to that of untreated samples. Meanwhile, the up-regulation of the *SS* gene was lower, i.e., it increased 12-fold. The quantitative RT-PCR analysis confirmed the up-regulation of the *DS* gene, which was the most intense after 24 h of treatment with 25 µM of carvacrol. Furthermore, the performed analyses showed the lowest (10-fold) increases in the maximum relative expression of the *DS* gene in comparison to this parameter estimated for *FPS* and *SS*.

### 2.2. Effect of Carvacrol Treatment on Ginsenoside Production in Hairy Roots of P. quinquefolium

In hairy root cultures of *P. quinquefolium* cultivated in shaken flasks, the total content of ginsenosides and the levels of protopnaxadiol and protopanaxatriol derivatives were estimated after 24 h and 72 h of treatment with different carvacrol concentrations (5, 10, 25, 50, 100, 250, and 500 μM). Moreover, the level of eight individual metabolites—Rb1, Rb2, Rb3, Rc, Rd, Rg1, Rg2, and Re—was examined.

**Figure 4:**
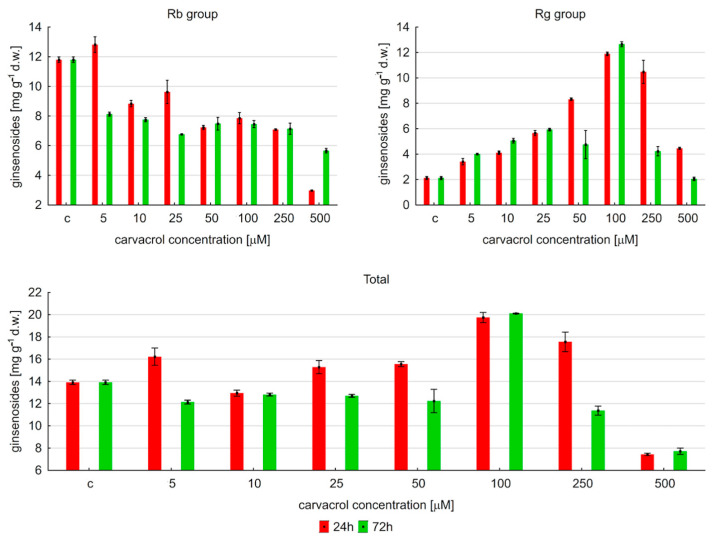
The content of ginsenosides depending on carvacrol concentration in *P. quinquefolium* hairy root cultures, “c” means control sample.

This study showed that carvacrol induced changes in the total content of saponins depending on the time of exposure and the concentration of carvacrol. A shorter elicitation (24 h) with carvacrol at 5–50 μM resulted in a slight increase (by about 10%) in ginsenoside content compared to the control samples (Figure 4). In contrast, longer exposure with carvacrol at 5–50 μM resulted in a slight decrease (also by about 10%) in saponin accumulation. The highest level of these metabolites was observed with 100 μM of carvacrol for both times of treatment, and its values were 19.75 and 20.01 mg∙g^−1^ d.w. (no statistically significant differences), respectively, after 24 and 72 h of elicitation. An amount of carvacrol higher than 100 μM reduced the saponin level for both times of elicitor treatment. At a 250 μM concentration of carvacrol, it was observed that the decrease was more intense after an extended time of exposure (72 h). The highest amount of carvacrol (500 μM) dramatically resulted in the depletion of the total saponins, regardless of the elicitation time. Then, their level was significantly lower than that determined in untreated samples.

The results of the experiments also showed that ginsenosides belonging to the Rb and Rg groups responded differently to carvacrol treatment. The accumulation of protopanaxadiol derivatives (expressed as the sum of Rb1, Rb2, Rb3, Rc, and Rd) was inhibited after carvacrol treatment, regardless of the concentration of the elicitor and the time exposure, except for 5 μM of carvacrol after 24 h of treatment. In contrast, the level of protopanaxatriol derivatives gradually increased with an increasing carvacrol concentration, until the maximum level was reached at 100 μM of carvacrol, and then the ginsenoside content gradually decreased to the level observed in the control. The highest content of protopanaxatriol (expressed as the sum of Re, Rg1, and Rg2) was achieved after 72 h of exposure (100 μM of carvacrol). The level was six-fold higher than that in untreated samples.

**Figure 5:**
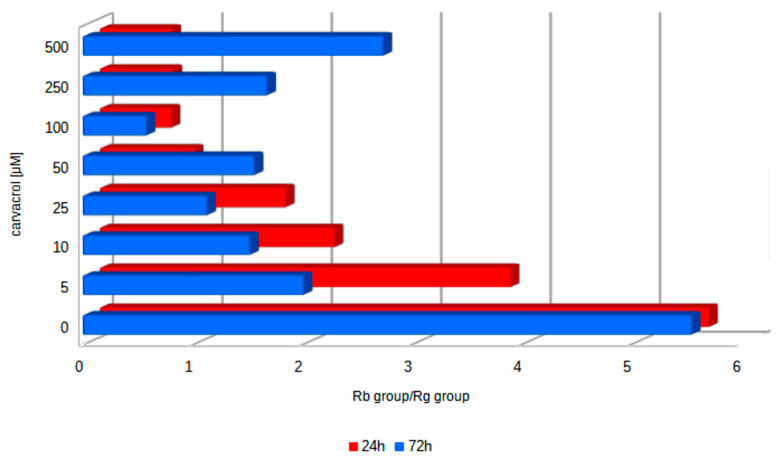
The ratio of total content of Rb-group ginsenosides/total content of Rg-group ginsenosides (RbG/RgG) in hairy root cultures of *P. quinquefolium*.

An estimation of the ratio of derivatives belonging to Rb-group ginsenosides and Rg-group ginsenosides (RbG/RgG) allows for the assessment of the quality of ginseng root. The obtained results (Figure 5) showed that this parameter was the highest (5.56) in the control samples. Its gradual reduction to 0.66 was found with an increasing carvacrol concentration after a shorter time exposure. The lowest level of RbG/RgG (0.59) was noted after 72 h of treatment with 100 μM of carvacrol, and it was more than nine-fold lower than the level in untreated samples. The observed changes in RbG/RgG levels indicated that the protopanaxatriol derivative content significantly increased after carvacrol elicitation.

**Figure 6:**
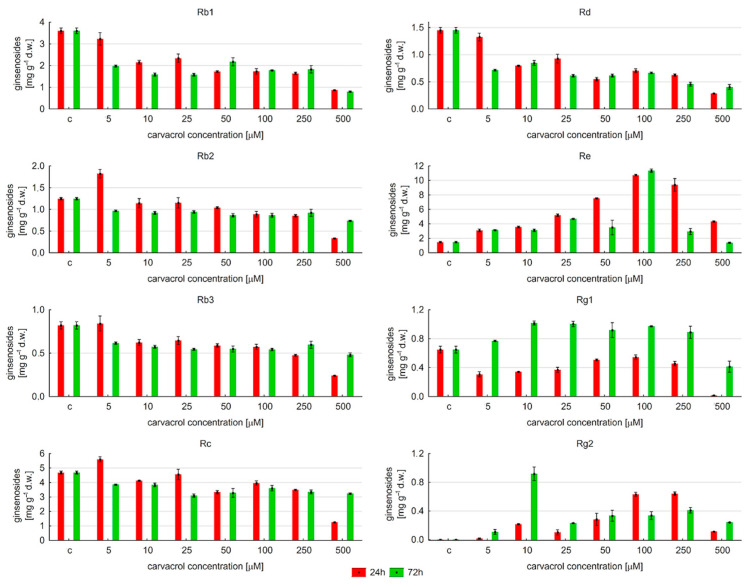
The effect of carvacrol treatment on the accumulation of individual ginsenosides in hairy roots of *P. quinquefolium*, “c” means control sample.

This research also determined the content of individual saponins and the profile of their change depended on the condition of carvacrol elicitation (Figure 6). Saponins Rc and Rb2 increased their level, respectively, by about 20% and 46% after 24 h of exposure with 5 μM of carvacrol. A higher concentration of carvacrol and a longer time of elicitation reduced the Rc and Rb2 content. Other results were noted in relation to compounds Rb1 and Rd. Their levels were reduced in comparison to those of the control samples, regardless of carvacrol concentrations and the time of exposure. After 24 h with 5 μM of carvacrol, they reduced by about 10%. The amount of both saponins decreased to approximately 50% of the value noted for the control, with an increase in the carvacrol concentration, and then remained constant (except for 500 μM of carvacrol). The lowest amount of Rb1 and Rd was noted for 500 μM of carvacrol. The accumulation of Rb3 was also inhibited, except for 5 μM of carvacrol and 24 h of elicitation. Then, its level was comparable to that observed in untreated samples. Moreover, it was noticed that after reduction to a specific level, the content of individual protopanaxadiol derivatives was maintained at a similar level for different concentrations of carvacrol, regardless of the time of elicitation (except for a concentration of carvacrol of 5 μM and 24 h of exposure).

In contrast, the level of Re saponins increased after carvacrol treatment. Regardless of the time of elicitation, the amount gradually enhanced with the carvacrol concentration, achieving its maximum level (11.34 mg∙g^−1^ d.w.; 72 h of elicitation) at 100 μM, and it was 7.72-fold higher in comparison to the control samples. The improved accumulation of the Rg1 metabolite was noted after 72 h of elicitation at 5–250 μM of carvacrol. Initially, its content increased by about 18.65% (5 μM). Its highest level was noted at 10–100 μM of carvacrol, i.e., 0.973–1.016 mg∙g^−1^ d.w. (approximately a 1.5–1.57-fold increase in comparison to the control). A higher concentration of carvacrol decreased the Rg1 level. Interesting results were obtained for the Rg2 metabolite. It was observed that the biosynthesis of Rg2 was induced after carvacrol treatment. Its maximum level (0.918 mg·g^−1^ d.w) was for 10 μM of carvacrol, after a longer time of elicitation (72 h). This compound was undetected in untreated samples.

**Figure A1:**
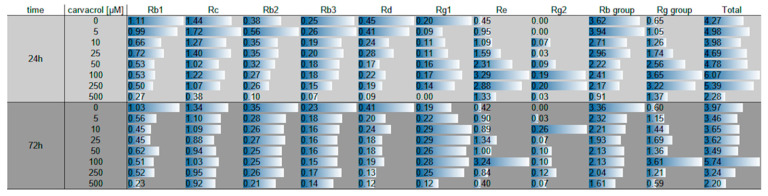
Productivity level of ginsenosides in hairy root cultures of *P. quinquefolium*.

The yield of ginsenoside biosynthesis was also characterized by a productivity evaluation of the studied cultures (Figure A1). The value of this parameter was 3.97 mg∙L^−1^∙day^−1^ in the control culture for all studied saponins. Their highest productivity was 5.74 mg∙L^−1^∙day^−1^ (100 μM of carvacrol and 72 h of elicitation). Among the individual studied metabolites, saponin Re showed the most enhanced productivity (an increase from 0.419 mg∙L^−1^∙day^−1^ in the control to 3.236 mg·L^−1^∙day^−1^ after carvacrol treatment with a 100 μM concentration and 72 h of elicitation).

**Figure 7:**
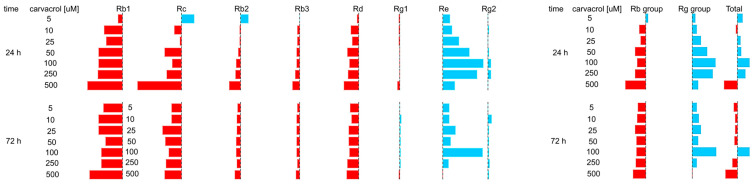
Ginsenoside productivity expressed as levels above (blue color) or below (red color) productivity in the control in hairy roots of *P. quinquefolium*.

**Figure A2:**
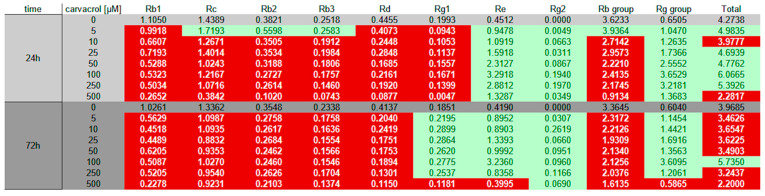
Value of ginsenoside productivity expressed as levels above (green color) or below (red color) productivity in the control in studied hairy root cultures.

Figure 7 and Figure A2 show the productivity expressed as levels above or below the productivity in the control. An analysis of the obtained results showed that metabolites, being protopanaxadiol derivatives, respond to carvacrol treatment by reducing their productivity below the level determined for the control (except Rc and Rb2, with 24 h of exposure to 5 µM of carvacrol). Moreover, it was noticed that carvacrol treatment resulted in the high stimulation of Re biosynthesis and the induction of Rg2 production. The weakest influence of carvacrol was observed for the productivity of the Rg1 compound. In addition, Figure 6 demonstrates similar changes in the profile productivity of the Rg group and saponin Re and shows that they depend on the carvacrol concentration and the time of exposure.

Moreover, the analysis of the Spearman’s rank correlation coefficient for relationships between the ginsenoside level and the carvacrol concentration in two time points of exposure shows a high positive correlation for saponins of the Re, Rg2, and Rg groups (0.72, 0.65, and 0.79, respectively) after 24 h of elicitation of carvacrol (Table 1). A negative correlation was stated in relation to all compounds belonging to the Rb group, regardless of the time of elicitation. These results indicated a strong relationship between the content or productivity of most studied saponins and the carvacrol concentration, especially for a shorter time of treatment.

## 3. Discussion

Most reports concerning essential oils (EO) are focused on research related to their chemical profile and biological activities [35]. One of the interesting examples of EO ingredients is carvacrol (2-Methyl-5-[1-methyl ethyl]-phenol). It is a naturally occurring phenolic monoterpenoid and a cymene derivative, present in the essential oils of *Thymus vulgaris*, *Lepidium flavum*, *Origanum scabrum*, *Origanum microphyllum*, *Origanum onites*, and *Origanum vulgare.* In vitro and in vivo studies confirmed pharmacological activities of carvacrol such as antidiabetic action and antioxidant, anti-inflammatory, hepatoprotective, antitumor, neuroprotective, and antimicrobial properties [36,37].

Bearing in mind that EO compounds demonstrate multidirectional activity and the fact that there are no studies on the application of essential oil ingredients to intensify the accumulation of secondary metabolites in plants, our team started investigating the influence of essential oil components on the biosynthesis of medicinal metabolites in hairy root cultures [33,34]. This study is a continuation of previous research and the first one on carvacrol application to enhance triterpene saponin production in *P. quinquefolium*-transformed root cultures and carvacrol influence on gene expressions’ coding of key enzymes of the ginsenoside pathway.

The obtained results revealed that the maximum total content of the tested saponin was about 20 mg∙g^−1^ d.w. (19.75 mg∙g^−1^ d.w. and 20.01 mg∙g^−1^ d.w., respectively, after 24 and 72 h of elicitation) with 100 μM of carvacrol. This amount was similar to the level observed for all ginsenosides after the yeast extract elicitation of *P. quinquefolium* hairy root cultures [29]. In contrast, methyl jasmonate treatment contributed to a higher total content of ginseng saponin [30]. Transformed root cultures of American ginseng were also elicited with use of linalool and *trans*-anethole [33,34]. Then, it was also noticed that these ingredients of EO stimulated ginsenoside accumulation in *P. quinquefolium* cultures.

The optimal concentration and exposure time of essential oil compounds to stimulate secondary metabolite accumulation should be experimentally estimated [33,34]. Carvacrol, similarly to *trans*-anethole and linalool, improved the total ginesenoside accumulation after short-time elicitation, whereas methyl jasmonate brought about the most intensive biosynthesis of ginseng saponin after 7 days of treatment in these cultures and in hairy root cultures of another species of ginseng—*Panax ginseng* [18,30,33,34]. Moreover, Palazón et al. [19] and Langhansova [38] showed significant differences among the used elicitors in ginsenoside production in hairy and adventitious root cultures of Korean ginseng, respectively. In addition to the elicitor type, its concentration is also highly important in ensuring a successful elicitation process. The results of this study showed that different concentrations of carvacrol allow for achieving the maximum contents of individual ginsenosides. A similar observation was noticed after linalool elicitation [33]. In contrast, less varied findings, especially regarding protopanaxadiol derivatives, were obtained in response to the *trans*-anethole and methyl jasmonate treatment of *P. quinquefolium* hairy root [30,34]. Elicitation with carvacrol reduced Rb1, Rb3, and Rd levels in studied cultures. A similar effect was noticed after linalool treatment, in relation to saponin Rb1 [33]. The decreased production of some Rb group ginsenosides was also observed in different phenotypes of *P. ginseng* hairy root cultures’ treatment, depending on the type of applied elicitors [19]. The stimulant influence of carvacrol (at 10–100 μM) was noticed for Rg1, Rg2, and Re saponins. A significant increase in the level of Rg1 and Re ginsenosides was noted after *trans*-anethole and methyl jasnonate treatment. Then, the Rg2 metabolite was undetected [30,34], while the study on *P. quinquefolium* hairy root cultures, elicited with abscisic acid, demonstrated an increased Rg2 level [31].

The change in individual ginsenoside contents influences the RbG/RgG parameter. In studied cultures, this parameter was 5.56 in the control samples, and its level was the lowest, 0.59, after carvacrol treatment (72 h of treatment with 100 μM of carvacrol), which implies that the share of protopanaxatriol derivatives significantly increased in the entire pool of determined compounds. These results differed from the ones obtained after the yeast elicitation of *P. quinquefolium* hairy root cultures [29]. In the transformed root of *P. ginseng*, the ratio of derivatives belonging to Rb-group ginsenosides and Rg-group ginsenosides ranged between 0.28 and 0.38 in the control and oscillated between 0.4 and 0.81 depending on the phenotype of the hairy roots and the type of elicitor during 72 h of exposure [19].

In addition to content, productivity also indicates that a particular culture can be potentially applied on an industrial scale. An analysis of productivity revealed that its value increased from 3.97 mg∙L^−1^∙day^−1^ in the control to the maximum, i.e., 5.74 mg∙L^−1^∙day^−1^, after carvacrol treatment (100 μM of carvacrol and 72 h of elicitation). *P. quinquefolium* hairy root cultures, growing in shaken flasks and elicited with methyl jasmonate, demonstrated a similar level of productivity. This parameter was, however, lower in comparison to the parameter observed in bioreactor cultures [30]. In the case of adventitious and hairy roots of *P. ginseng,* the productivity values were 1 mg∙g^−1^ d.w.∙L^−1^∙day^−1^ and 1.9 mg∙g^−1^ d.w.∙L^−1^∙day^−1^ (cultures cultured in flasks), respectively [19,39]. They were significantly lower in comparison to the productivity levels in samples of *P. quinquefolium* hairy root cultures untreated and treated with carvacrol and described in this paper.

**Figure 8:**
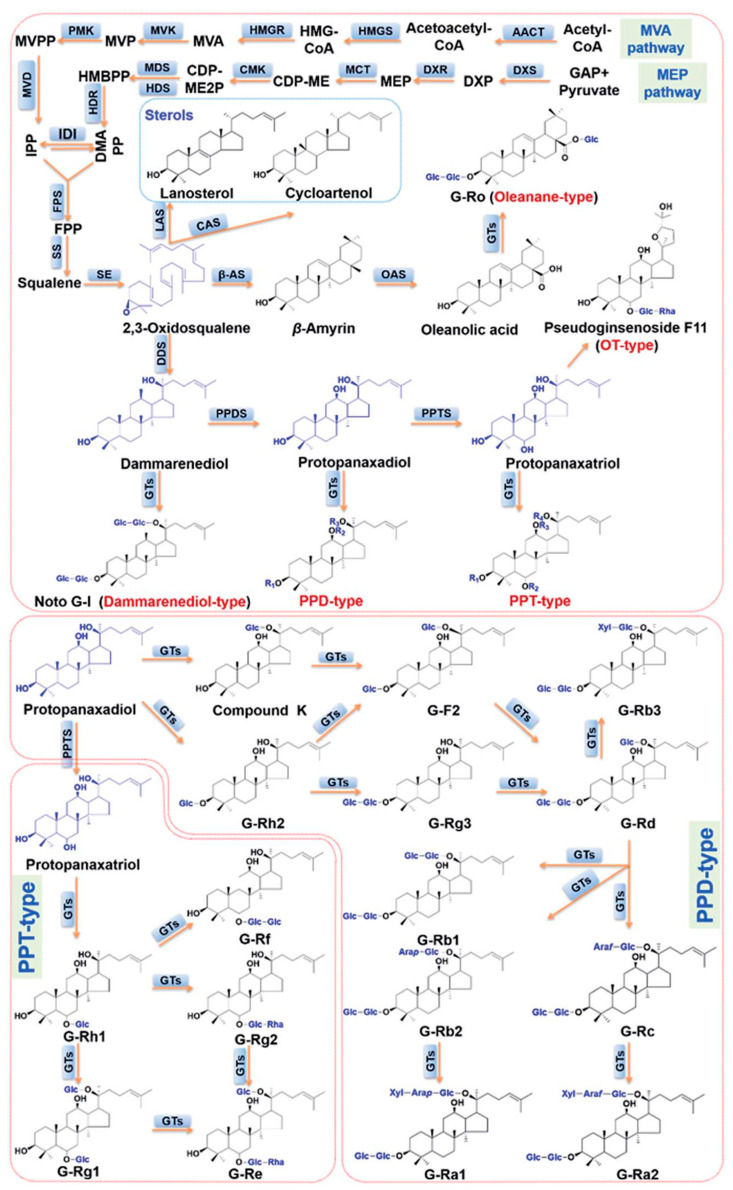
Scheme of the ginsenoside biosynthetic pathway in ginseng according to Li et al. [42]. ACT—acetyl-CoA acetyltransferase; CAS—cycloartenol synthase; CDP-ME—4-cytidine diphosphate-2-C-methyl-D-erythritol; CDP-ME2P—CDP-ME-2-phosphate; CMK—CDP-ME kinase; DDS—dammarenediol synthase; DMAPP—dimethylallyl pyrophosphate; DXP—1-deoxy-D-xylulose-5-phosphate; DXR—reductoisomerase; DXS—DXP synthetase; FPP—farnesyl diphosphate; FPS—farnesyl diphenylphosphate synthase; GAP—glyceraldehyde-3-phosphate; GTs—glycosyltransferases; HDR—HMBPP reductase; HDS—HMBPP synthetase; HMBPP—(E)-4-hydroxy-3-methylbut-2-enyl diphosphate; HMG-CoA—3-hydroxy-3-methylglutaryl-CoA; HMGR—HMG-CoA reductase (3-hydroxy-3-methylglutaryl-CoA reductase; HMGS—HMG-CoA synthase; IDI—isopentenyl diphosphate isomerase; IPP—isopentenyl diphosphate; LAS—lanosterol synthase; MCT—4-cytidine diphosphate-2-C-methyl-D-erythritol synthetase; MDS—2-C-methyl-D-erythritol-2,4-cyclic phosphate synthetase; MEP—2-C-methyl-D-erythritol 4-phosphate; MVA—mevalonic acid; MVD—mevalonate diphosphate decarboxylase; MVK—mevalonate kinase; MVP—mevalonate phosphate; MVPP—mevalonate diphosphate; OAS—oleanolic acid synthase (CYP716A52); PMK—phosphomewalonate kinase; PPDS—protopanaxadiol synthase (CYP716A47); PPDS—protopanaxadiol synthase (CYP716A47); PPTS—protopanaxatriol synthase (CYP716A53); SE—squalene epoxidase; SS—squalene synthase; β-AS—β-amyrin synthase.

An analysis of parameters characterizing the ability of hairy root cultures of *P. quinquefolium* to produce ginsenoside indicated that carvacrol can affect the biosynthesis of ginseng saponins. Additionally, the obtained results demonstrated that carvacrol can affect the gene expression level. It up-regulated the expression of three genes, *FPS*, *SS*, and *DS*, involved in ginsenoside biosynthesis. The *FPS* gene codes farnesyl pyrophosphate synthase (FPS). This enzyme performs a pivotal role in the branch point in the MVA pathway (Figure 8). Dimethylallyl pyrophosphate (DMAPP) and 3-isopentenyl pyrophosphate (IPP) are substrates of farnesyl pyrophosphate synthase. DMAPP and two units of IPP undergo a condensation reaction using FPS, which leads to the formation of farnesyl pyrophosphate. These processes include two steps. At first, geranyl diphosphate (GPP) is obtained, and, then, GPP is converted to farnesyl diphosphate (FPS) [40]. Carvacrol contributed to a maximum (70-fold) increase in *FPS* gene expression in hairy root cultures of *P. quinquefolium* at a 10 μM concentration and with 12 h of treatment. Similarly, within 12 h a clearly up-regulated *FPS* gene was observed by Kim et al. [18] in the transformed root culture of *P. ginseng* elicited with methyl jasmonate. Another investigation showed that MJ treatment enhanced the mRNA level of *P. ginseng FPS* within 12 h of treatment, achieving its maximum value after 24 h. In consequence, the increased activity of farnesyl diphosphate synthase was observed between 12 h and 7 days of methyl jasmonate elicitation [41]. Moreover, transgenic lines of *P. ginseng* root, obtained from the adventitious root by inserting the *P. ginseng FPS* gene that regulated the activity of farnesyl diphosphate synthase, showed an 11.1-fold higher level of the transcriptions of that gene and a 2.4-fold increase in the ginsenoside content in comparison with the control [26].

The results of this paper confirmed that the expression of the *SS* gene was also up-regulated after carvacrol treatment, reaching its maximum 12-fold increase in comparison to the control. This observation confirmed that carvacrol exhibits a weaker effect on the *SS* gene than on the *farnesyl pyrophosphate synthase* gene. The *squalene synthase* gene codes squalene synthase (SS), which carries out the reaction leading to squalene formation by condensing two molecules of farnesyl diphosphate. Squalene is considered a precursor of 2,3-oxidosqualene and dammarenediol [40]. The important role of the stimulation of *SS* gene expression, in order to ensure effective ginsenoside production with the use of the elicitation process, was demonstrated in many previous reports [18,27,43,44]. The response of the hairy root of *P. quinquefolium* was extremely strong and manifested with the up-regulation of the *squalene synthase* gene (a 2435.4-fold increase in expression in comparison to the control) after MJ elicitation. In consequence, the production of saponin was significantly enhanced [30]. The influence of methyl jasmonate on *SS* gene expression was also tested in root cultures of *P. ginseng* [27], and it was observed that the up-regulation of the *SS* gene increased the expression of downstream genes, such as the *squalene epoxidase* gene *(SE)*. Furthermore, the increased production of ginsenosides belonging to dammaran-type metabolites was noticed. This information implied that the *SS* gene can take part in the regulation of a further stage of the ginseng saponin biosynthetic pathway.

Moreover, some data demonstrated a significant improvement of the expression of *Panax ginseng* genes, such as *HMGR*, *geranyl diphosphate synthase (GPS)*, *FPS*, *SS*, and *SE*, after fungal elicitor treatment, which resulted in the enhanced production of ginseng saponins [45].

Dammarenediol is formed as a result of squalene transformation to 2,3-oxidosqualene. Next, 2,3-oxidosqualene is cyclized to a tetracyclic dammarenediol backbone. Cyclization reaction occurs with the use of dammarenediol-II synthase (DS). Our result demonstrated the up-regulation of the gene coding dammarenediol-II synthase. What is interesting is the fact that there are reports that confirm a relationship between the overexpression of the *DS* gene and the enhanced production of ginsenosides in *P. ginseng* [40,46,47]. The formation of dammarenediol is an important element of ginsenoside biosynthesis, considering the fact that dammarenediol, via oxidation, hydroxylation, and glycosylation, can be converted to dammaran-type ginsenosides [48].

The results of this paper showed enhanced *FPS*, *SS*, and *DS* expression and improved ginsenoside accumulation. Our findings also demonstrated that carvacrol affected the production of protopanaxadiol and protopanaxatriol derivatives in a different way. It can be concluded that the accumulation of saponins belonging to the Rb group decreased, whereas the accumulation of saponins belonging to the Rg group increased. This effect could be associated with the different activation of protopanaxadiol synthase (PPDS, CYP716A47) and protopanaxatriol synthase (PPTS, CYP716A53v2). Both the enzymes are key for the formation of PPD and PPT aglycones. In addition, CYP716A53v2 is known to enable the conversion of PPD-type ginsenosides into PPT-type ginsenosides [49,50]. Park et al. [51] indicated that the overexpression of *PPTS* in hairy roots cultures of *P. ginseng* contributed to the enhanced accumulation of *PPTS* mRNA in transgenic roots compared to the control—nontransgenic roots. In contrast, the silencing of *PPTS* mRNA in RNA reduced *PPTS* transcription [51].

The biosynthesis of individual ginsenosides is possible via glycosylation, performed by UDP-dependent glycosyltransferases (UGTs) (Figure 8). UGTs form a glycosidic bond using sugar moieties [52]. Up to now, UGTs, involved in ginsenoside biosynthesis, have not been fully recognized [53,54,55,56,57,58]. In *P. ginseng*, UDP-dependent glycosyltransferases add monosaccharides to triterpene aglycones, mainly at positions C-3 and/or C-20 in PPD-type ginsenosides or C-6 and/or C-20 in PPT-type ginsenosides [59]. Sun et al. [58] showed that MJ treatment up-regulated 11 UGTs and down-regulated 3 UGTs in American ginseng. The results of our study revealed a significant increase in the Re metabolite content and the induction of Rg2 biosynthesis as well as the reduced accumulation of Rb-group saponin. Hence, we can conclude that carvacrol possibly influences the regulation of UGT genes and, in consequence, UDP-dependent glycosyltransferases activity in studied cultures of *P. quinquefolium* hairy root. The ginsenoside biosynthesis pathway proposed by Li et al. [42] and Hou et al. [52] shows that the Rg1 metabolite is a substrate for Re biosynthesis. In addition, Rg2 saponin can be generated via the conversion of Rh1 ginsenoside with the use of a suitable glycosyltransferase. In light of the knowledge concerning ginsenoside biosynthesis and the obtained result of individual metabolite contents, it seems possible to make a hypothesis about the potential stimulating influence of carvacrol on glycosyltransferases’ activity, converting metabolite Rg1 to Re, regulating Rg2 generation and inhibiting its influence on glycosyltransferases activity, and leading to the obtainment of Rd, Rb1, Rb2, or Rb3 saponins. However, the potential influence of carvacrol on glycosyltransferases, which are involved in ginsenoside biosynthesis, will have to be verified in further studies after identifying these enzymes and preparing their detailed characteristics.

The obtained results also indicated that the tested overexpression genes were achieved at different concentrations of carvacrol than the maximum level of the studied metabolites. This investigation proved that carvacrol up-regulated *FPS*, *SS*, and *DS* genes. Probably, other factors play an important role in the regulation of ginsenoside biosynthesis. It should be noted that the expression of genes encoding enzymes of the last stage of biosynthesis, e.g., protopanaxadiol synthase or glycosyltransferases, in response to carvacrol has not been examined. Thus, the effect of carvacrol is not known in relation to these genes. The reasons are the imprecise recognition of these genes for *P. quinquefolium* and the lack of information about them in the gene bank. In addition, little is known about the effect of transcription factors on the regulation of ginseng saponin biosynthesis at the molecular level.

In this research, the decrease in the content of some saponins was observed after 72 h of elicitation, compared to their level after 24 h of carvacrol treatment. This may probably be due to the inhibition of the expression of genes encoding enzymes involved in the biosynthetic pathway. This effect was observed for FPS and SS. It was found that elicitation lasting longer than 12 h resulted in a decrease in the expression level of these genes. A similar mechanism may apply to genes encoding enzymes of the last stage of ginsenoside biosynthesis. In contrast, enzyme activities can also be metabolically controlled. This may, especially, apply to glycosyltransferases, which ultimately form individual metabolites. However, there is no detailed characterization of these enzymes in *P. quinquefolium*.

Bearing in mind the current state of knowledge, it is impossible to complete an explanation of the mechanism of carvacrol action in ginsenoside biosynthesis, so further studies are necessary.

## 4. Materials and Methods

### 4.1. Hairy Roots of P. quinquefolium Cultured in Shaken Flasks

Hairy roots of *P. quinquefolium* (ca. 0.30 g fresh weight and 28.0 mg dry weight) were placed in a shaken flask filled with 80 mL of modified medium B-5 [60,61]. The root biomass was cultivated in the dark, in rotary shaker (100 rpm) at 26 °C ± 2 °C.

### 4.2. Elicitation of Hairy Root Cultures of P. quinquefolium

Carvacrol (Sigma-Aldrich, St. Louis, MO, USA) was dissolved in 96% ethanol and added to the medium by sterile syringe filter Millex GS 0.22 µm (Millipore, Burlington, MA, USA) at concentrations of 5, 10, 25, 50, 100, 250, and 500 µM. Carvacrol was added to the culture on day 25 of the growth cycle. The exposure time of elicitation was 24 and 72 h. The control sample contained ethanol instead of carvacrol.

### 4.3. RNA Isolation and Reverse Transcriptase (RT)-PCR Analysis

*P. quinquefolium* hairy root cultures after 6, 12, and 24 h of carvacrol elicitation were used for total RNA isolation. The Total RNA Prep Plus Minicolumn Kit (A&A Biotechnology, Gdańsk, Poland) was used for RNA isolation according to manufacturers’ protocol, and details were provided in a previous article [34]. The High-Capacity cDNA Reverse Transcription Kit (Applied Biosystems; Thermo FisherScientific, Inc., Waltham, MA, USA) was used to process the obtained RNA into cDNA. The composition of reaction mixture was as follows: dNTPs (final concentration 1 mM), anchored oligo (dT)23 (final concentration 3.5 μM), 2 μL of 10× buffer, RNase inhibitor (20 U/μL), and MultiScribe Reverse Transcriptase (RT; 50 U/μL). DNaseI digestion was performed on all RNA samples, and this was verified by quantitative real-time PCR reaction with control samples of RNA without the standard reverse-transcription step. The cDNA was immediately used or stored at −20 °C.

### 4.4. Qualitative Analysis

Nucleotide sequences of primers, used for qualitative analysis, are presented in Table 2. The “OligoCalc: an online oligonucleotide properties calculator” (http://biotools.nubic.northwestern.edu/OligoCalc.html, (accessed on 10 January 2023)) Kibbe [62] program was applied for primer design.

The reaction mixture used for PCR amplification comprised cDNA template, 0.5 μM of each primer, DreamTaq Green PCR Master Mix (2X) (Thermo Scientific), and PCR-grade water to the final volume of 25 μL. A negative control (sample without a cDNA template) was included in each experiment [30].

Qualitative PCR amplification steps were as follows: initial denaturation at 94 °C for two minutes, 35 cycles of denaturation at 94 °C for 15 s, primer annealing at 57 °C for 45 s, extension at 72 °C for 45 s, and the final extension at 72 °C for three minutes. The electrophoresis of PCR products was performed under condition described in a previous paper [30] with a 100 bp DNA ladder on 1.2% TBE-agarose gel (Bioline, London, UK).

### 4.5. Quantitative Analysis by Real-Time PCR

CFX Connect Real-Time PCR Detection System (Bio-Rad, Hercules, CA, USA) was used for quantitative expression analysis for all cDNA samples. The investigated genes and the reference gene (used as internal positive control and gene expression normalizer) were tested in triplicate and parallel for each sample in separate wells during the same reaction. Reaction mixture for each run consisted of 5.0 μL iTaq Universal SYBR Green Supermix reagent kit (Bio-Rad, Hercules, CA, USA), 0.7 μL forward and reverse primer (final concentration 0.2 μM), 2.6 μL nuclease-free water, and 1.0 μL template cDNA. The thermal cycling conditions were as follows: initial denaturation at 95 °C for two minutes and 40 cycles at 94 °C for 15 s and at 57 °C for 30 s. In order to verify product specificity, melting curve analysis was performed after the qPCR run. The efficacy of reaction for all tested genes was almost 100%, and the 2^−ΔΔCT^ method designed by Livak and Schmittgen [63] was used to calculate relative changes in *FPS*, *SS*, and *DS* gene expression in *P. quinquefolium*. Gene expression results were analyzed by CFX Maestro Software version 2.2 for CFX Real-Time PCR Instruments (Bio-Rad, Hercules, CA, USA). The one-way ANOVA method with STATISTICA 13.1 software (TIBCO, Palo Alto, CA, USA) was used to determine significant differences between investigated samples at *p* value < 0.05 [64].

### 4.6. Determination of Ginsenoside Content

#### 4.6.1. Sample Preparation

Dried hairy roots (1 g) were extracted three times using 80% methanol for 30 min. The extracts were combined and evaporated to dryness. Next, the extracts were purified with the use of an SPE column with octadecyl (C18). After that, the samples were quantified [29].

#### 4.6.2. Quantitative Analysis of Ginsenosides with the HPLC Method

Eight ginsenosides, Rb1, Rb2, Rb3, Rc, Rd, Re, Rg1, and Rg2 (all purchased from Aldrich Sigma, Darmstadt, Germany), were quantitatively analyzed according to Kochan et al. [34]. Briefly, HPLC analyses were performed on a C18 column (150 × 4.6 mm, 5 μm) using gradient elution: 0–16 min: 18% A (acetonitrile (J.T. Baker, Deventer, The Netherlands), 82% B (water, J.T. Baker, Deventer, The Netherlands); 17–28 min: 30% A, 70% B; 29–60 min: 32% A, 68% B; 61–64 min: 80% A, 20% B; 65–68 min: 18% A, 82% A. The flow rate was 2 mL min^−1^. Detection was carried out at 203 nm. The quantitative content of ginsenosides was set by comparing the retention time and peak areas between standards and samples.

The determined amounts of analyzed metabolites were presented as “content” (expressed in mg∙g^−1^ d.w.) and “productivity” (expressed in mg∙L^−1^∙day^−1^).

### 4.7. Statistical Analysis

All treatments procedures were performed in three independent experiments. The statistical analysis was carried out using the Kruskal–Wallis test. Any relationships were considered significant for *p* ≤ 0.05. Statistica Version 13.1 software was used for all statistical analyses (STATSoft, Tulsa, OK, USA).

## 5. Conclusions

This report demonstrated that carvacrol can play the role of elicitor in *P. qunquefolium* hairy root cultures. In addition, the obtained results revealed that carvacrol can stimulate the expression of *FPS*, *SS*, and *DS* genes, coding key enzymes involved in the ginsenoside pathway depending on the time of exposure and concentration. A quantitative analysis of the ginsenoside content usually indicated that carvacrol inhibited the accumulation of protopanaxadiol derivatives and stimulated the amount and productivity of another group of saponins: protopanaxatriol derivatives. It was noticed that carvacrol treatment maximally stimulated Re biosynthesis and induced Rg2 production—its maximum level was higher in comparison to the other elicitators used for enhanced ginsenoside accumulation in *P. quinquefolium* hairy root cultures.

## Figures and Tables

**Table 1:** Spearman’s rank correlation coefficient between ginsenoside content/productivity and carvacrol concentration and time of elicitation.

Ginsenosides	Elicitation 24 h	Elicitation 72 h
Rg1	−0.25	−0.06
Re	0.72	0.04
Rg2	0.65	0.47
Rb1	−0.91	−0.57
Rc	−0.83	−0.59
Rb2	−0.81	−0.69
Rb3	−0.82	−0.61
Rd	−0.90	−0.87
Rg group	0.79	0.08
Rb group	−0.87	−0.74
Total	0.09	−0.40

**Table 2:** Primers used for RT-PCR analysis.

Gene	Primers	GenBank Accession Number
*DS*	Forward: 5′CACAGCTGAAGCGCTAAAGT3′ Reverse: 5′ATATGGTTTTGGAACTGGAGGCT3′	KC316048.1
*FPS*	Forward: 5′ATAGATTTGATCACCACCCTT G3′ Reverse: 5′AGTATGTTTCTCCAGATCTTCGC3′	GQ401664.1
*SS*	Forward: 5′ATGCTGAAGTCCAAGGTTGAC3′ Reverse: 5′ATTGTATCCTGACTCATTCTCG3′	AM 182456.1
*actin*	Forward: 5′AAGCCCAATCGAAGAGAGGTA3′ Reverse: 5′ATCTTCTCCCTGTTGGCCTT3′	KF 699319.1

*DS*—gene coding synthesis of dammarenediol synthase; *FPS*—gene coding synthesis of farnesyl diphosphate synthase; *SS*—gene coding synthesis of squalene synthase; *actin*—reference gene coding synthesis of actin.

## Data Availability

Data are contained within the article.
